# A Cross-Sectional Study Evaluating the Basic Course in Biomedical Research Among Medical Postgraduates and Teachers

**DOI:** 10.7759/cureus.63551

**Published:** 2024-07-01

**Authors:** Ashwini P Singh, Vinay K Sayeli, Sadhana Gera, G Hari J Rao

**Affiliations:** 1 Department of Pharmacology, Alluri Sitarama Raju Academy of Medical Sciences, Eluru, IND

**Keywords:** mooc, swayam, teaching faculty, research methodology course, bcbr course

## Abstract

Introduction and methodology

This study aims to give feedback on the Basic Course in Biomedical Research (BCBR) program, an initiative of the National Medical Council (NMC) in collaboration with the National Institute of Epidemiology (ICMR-NIE) using the SWAYAM ("Study Webs of Active-Learning for Young Aspiring Minds") portal. The objectives of the study areto evaluate the usefulness of BCBR and the content and conduct of the BCBR program using a questionnaire among medical postgraduates and teachers. The study design used was a cross-sectional study and collected data from 392 participants who completed the BCBR program. A validated questionnaire was utilized to gather information on participant demographics, reasons for enrolment, satisfaction with course material, assignments, technical issues during exams, and perceived usefulness of the course.

Results

The study found a predominance of postgraduate students among participants (65.31%), reflecting their inclination towards research. Primary reasons for enrolment included institutional requirements and eligibility criteria to appear for the final postgraduate examination. Most participants utilized both videos and lecture hand-outs. This course facilitated its participants to improve their ability to conduct research by 89.03%. Feedback suggested a need for improvement in course content, particularly in simplifying statistical concepts and incorporating more interactive sessions and practical demonstrations.

Conclusion

The findings highlight the effectiveness of the BCBR program to up-skill the ability of participants to perform research. Overall, the study offers valuable feedback about the BCBR course.

## Introduction

Research on health fuels both scientific advancements and educational progress in healthcare [1]. India, a nation on the fast track to development, has witnessed a remarkable surge in health research endeavours and publications in recent years. This upward trajectory reflects a growing commitment to understanding and addressing health challenges unique to the country's vast and diverse population [2].

Quality medical research by teachers is not just about academic accolades. It strengthens patient care with new insights, enhances medical education for future generations, saves costs by optimizing treatments, and ultimately improves the health and well-being of society [3]. When it comes to scientists and universities, the quality of their research is like the gold standard - peers use it to measure their worth. However, research quality in Indian medical colleges and scientific institutions falls short of expectations in the eyes of their peers [4]. With the Medical Council of India's 2009 policy linking publications to promotions, medical teachers in India have faced a paradigm shift, evolving into hybrid academics tasked with both teaching and research excellence.

In a groundbreaking move, the National Medical Commission (NMC) has made the Basic Course in Biomedical Research (BCBR) mandatory for medical and dental postgraduates and teachers across India. This online course equips healthcare professionals with essential research skills, ensuring a future of evidence-based practice and advancements in healthcare [5].

Recognizing its expertise in online health research education, the National Medical Council (NMC) appointed the National Institute of Epidemiology (ICMR-NIE) to develop and deliver a nationwide online course on research methodology for medical and dental postgraduates and faculty. ICMR-NIE's proven track record in creating massive open online courses (MOOCs) through the Indian Ministry of Education's SWAYAM platform ("Study Webs of Active-Learning for Young Aspiring Minds") underpinned their selection. Leveraging this experience, ICMR-NIE designed and delivered the "Basic Course in Biomedical Research" (BCBR) on SWAYAM [6].

The BCBR adhered to a four-quadrant instructional design model, which was prepared by subject experts, encompassing e-tutorials (video lectures), e-content (reading materials, lecture hand-outs, and text transcripts), a discussion forum, and assessments. The curriculum consisted of 23 lectures, each approximately 20 minutes in duration. With this model, BCBR covered a broad spectrum of topics related to research methodology areas such as research conceptualization, epidemiological and bio-statistical considerations in research design, research planning and execution, research protocol writing, and publication ethics.

Feedback is very important to drive any process. Like the positive and negative feedback cycles in biology, effective feedback acknowledges the positive impact, and it highlights the areas that need improvement. Ultimately, feedback drives improvement. It fuels refinement and offers better solutions.

The feedback about the course is collected using a validated questionnaire. Validity ensures that a study measures what it intends to measure, accurately capturing the specific area of investigation. Four types of validity can be considered for evaluating a questionnaire - they are face validity, content validity, construct validity, and criterion validity.

Face validity is the weakest form of validity. This evaluation is captured from non-subject experts.

Content validity encompasses an exhaustive literature search to include the relevant items. After that, a survey questionnaire needs to be generated, and each item in the survey questionnaire is assessed using a three-point Likert scale. The survey should be sent to the experts in the area of research intended. The content validity ratio (CVR) is then calculated for each item by employing the Lawshe method [7].

Construct validity can be applied if the relationship between various domains is causal (cause and effect relationship).

Criterion or concrete validity describes the extent to which a measure is related to an outcome. It measures how well one measure predicts an outcome for another measure.

Content validity is a highly recommended method and appropriate method for the questionnaire that has been developed for this study. The validity of the questionnaire was assessed using CVR analysis.

The authors of the article after completing the BCBR course felt the need for giving collective feedback from the people who have completed this course. As graduates of the BCBR course, the authors of this article personally felt the need to express thanks for all the efforts that were kept by NMC and ICMR-NIE in designing this course, offering it for free and the price was only collected to conduct the exam, if the person wants to get certified. At the same time, the authors have not found many articles evaluating this course. Thus, this study is designed to give feedback on the BCBR course using a questionnaire.

The objectives of this study are as follows: to evaluate the usefulness of the BCBR program among medical postgraduate students and teachers using a questionnaire and to evaluate the content and conduct of the BCBR program among medical postgraduate students and teachers using a questionnaire.

## Materials and methods

Study design

The design used for this research proposal was a cross-sectional study design. A web-based questionnaire was developed, validated, and circulated using Google Forms to collect the data. Personal details such as name and institute affiliations were not captured to anonymize the details of the submission.

Study population

The study included medical postgraduate students and teachers who enrolled in the BCBR program, successfully completed the exam, and were willing to participate in the research. Those who had not completed the BCBR exam or were unwilling to provide consent were excluded.

Ethical consideration

This study was initiated after receiving approval from the Institutional Ethics Committee approval letter (ASRAMS BHR-EC Approval no: 138/2023).

Study duration and sample size

The study was conducted over nine months (March 2023 to December 2023), and a total of 392 questionnaire forms from 392 subjects were collected during this period (the minimum sample size estimated was 385) [8], considering 50% of the population may say that this course as useful.

Methodology for the validation of the questionnaire

The questionnaire was sent to 15 subject experts. The essentiality of each question was assessed using a three-point Likert scale (not necessary, useful but not essential, and essential). The CVR was calculated using the following formula:

CVR=(ne- (N/2))/(N/2)

Where ne indicates the frequency of essential responses and N/2= 7.5 (i.e., 15/2). The questions with a greater than 0.5 CVR ratio were included in the questionnaire.

The final questionnaire had 14 close-ended questions, which were summarized in the results section and one open-ended question asking suggestions (if any) about the BCBR course.

Statistical analysis

Results were summarized descriptively. All the categorical variables were expressed in percentages.

## Results

Demographics of the study sample

Among study participants, 65.31% were postgraduate students, 3.83% were senior residents/tutors, 7.4% were assistant professors, 9.44% were associate professors, and 14.0% were professors (Table 1).

**Table 1:** Distribution of the sample

S. No	Postgraduate students	Senior residents/tutors	Assistant professors	Associate professors	Professors
Frequency	256	15	29	37	55
Percentage	65.31	3.83	7.40	9.44	14.00

Reason for enrolment

The study participants mentioned the following as their reason for enrolment in this course; 61.73% as it is mandatory for postgraduates to appear for the final exam, 19.39% as it is mandatory for teaching staff promotions, 17.09% out of self-interest, and 1.79% as it is free course with certificate (Table 2).

**Table 2:** Reason for enrolment

S. No	Free course with certificate	Mandatory for post-graduates to appear for the final exam	Mandatory for teaching staff promotions	Self-interest
Frequency	7	242	76	67
Percentage	1.79	61.73	19.39	17.09

Difficulty during enrolment into the course

Among the study participants, 47.7% felt that the enrolment process of the course was neither easy nor difficult, and only 1.79% felt it was very difficult (Table 3).

**Table 3:** Difficulty in enrolment

S. No	Very easy	Easy	Neither easy nor difficult	Difficult	Very difficult
Frequency	60	101	187	37	7
Percentage	15.31	25.77	47.7	9.44	1.79

Course material

The course material used by participants for completion of assignments was reported to be only videos by 3.83 %, only lecture hand-outs by 21.68%, and both videos and hand-outs were used by 74.49% (Table 4).

**Table 4:** Material used

S. No	Both	Only lecture handouts	Only videos
Frequency	292	85	15
Percentage	74.49	21.68	3.83

Satisfaction with the content provided in the course material

Among the study participants, 68.11% were satisfied with the content provided in the study materials, and 31.89% of participants felt the need to improve the existing content in the study material (Table 5).

**Table 5:** Satisfaction with the content

S.No	Can be improved	Yes
Frequency	125	267
percentage	31.89	68.11

Need for interactive sessions

Among the study participants, 77.55% reported that, if the interactive sessions were conducted during the course, it would have helped them for a better understanding of the subject (Table 6).

**Table 6:** Interactive sessions will be useful

S. No	No	Yes
Frequency	88	304
Percentage	22.45	77.55

Difficulty of assignments

Among the study participants, 49.74% expressed that it was neither easy nor difficult to complete the course assignments, whereas 20.66% and 1.79% population thought that it was difficult and very difficult, respectively (Table 7).

**Table 7:** Difficulty in completion of assignments

S. No	Very easy	Easy	Neither easy nor difficult	Difficult	Very difficult
Frequency	29	80	195	81	7
Percentage	7.4	20.41	49.74	20.66	1.79

Satisfaction with the guidance at the exam center

Satisfaction with the guidance provided at the exam center was found to be 66.58% (Table 8).

**Table 8:** Satisfied with the guidance at the exam center

S. No	Not satisfied	Satisfied	Somewhat satisfied
Frequency	23	261	108
Percentage	5.87	66.58	27.55

Technical glitches

Among the study participants, 88.78% had informed that they did not face any technical glitches during exams (Table 9).

**Table 9:** Question: Did you face technical glitches during exams?

S. No	No	Yes
Frequency	348	44
Percentage	88.78	11.22

Timeframe of assignments

Among the study participants, 67.09% expressed that the course will be more effective if there is a stipulated timeframe for completion of assignments (Table 10).

**Table 10:** Question: If there is a timeframe for completion of assignments and exams, do you think it will be more effective?

S. No	No	Yes
Frequency	129	263
Percentage	32.91	67.09

Pertinence of the questions asked in the exam

Almost 66.07% of study participants were able to correlate with the questions asked in the assignments with the course material (Table 11).

**Table 11:** Question: Do you think the questions asked in the exam were pertinent to the course assignments?

S. No	May be	No	Yes
Frequency	88	45	259
Percentage	22.45	11.48	66.07

Difficulty level of the exam

Among the study participants, 48.21% thought that the exam was neither easy nor difficult, ad 24.23% and 3.58% of participants expressed that it was difficult and very difficult, respectively (Table 12).

**Table 12:** Difficulty level of the exam

S. No	Very easy	Easy	Neither easy nor difficult	Difficult	Very difficult
Frequency	22	72	189	95	14
Percentage	5.61	18.37	48.21	24.23	3.58

Usefulness of the course

After completion of the course, 57.65% of the study participants mentioned that they learned data entry and statistical analysis (Table 13).

**Table 13:** Question: Were you able to learn data entry and statistical analysis by this course?

S. No	No	Yes
Frequency	166	226
Percentage	42.35	57.65

Among the study participants, 89.03% were able to carry out research in an effective way than before completing the course (Table 14).

**Table 14:** Question: Were you able to plan, understand, and perform the research in a better way after completion of the course?

S. No	No	Yes
Frequency	43	349
Percentage	10.97	89.03

## Discussion

The findings of this study shed light on various aspects of an educational course, including participant demographics, reasons for enrolment, perceived difficulty during enrolment, satisfaction with course materials, interactive sessions, assignments, technical issues during exams, and perceived usefulness of the course.

Among the study participants, 65.31 were postgraduate students, and the remaining were senior residents/tutors, assistant professors, associate professors, and professors. The predominance of postgraduate students' participation in the study reflects their attitude to participate in research projects. The students might find it easy to fill the Google Forms as they are more tech-savvy [9] (Table 1).

The reasons cited for enrolment into the course by the majority of the participants are the mandatory requirements for appearing in the final postgraduate examination and for the promotions of teaching faculty. This information reflects both institutional demands and individual career goals (Table 2).

The majority of participants used both videos and lecture handouts as the course material, as it seemed to be more beneficial than using a single mode of learning (Table 4).

The study conducted by Martin suggested that there is a significant shift towards visual content in academia and medical communications. The advantages of providing such videos are easy-to-grasp infographics that break down complex information to capture attention, enhance comprehension, and increase understanding and memory retention of the information [10].

The previous study done by Furnham et al. has shown that printed information can be as effective as videos. On repeated observation, they concluded that print is superior to non-interactive videos. Printed information also offers the advantage of quick revision. In this study, the majority of participants chose both methods rather than choosing one method and seized all the advantages of print materials and video content provided in the course [11].

In our survey, over 31.89% of participants expressed a need for improvement of the current content (Table 5). Their suggestions included that statistical terms should be explained in simple English, enhancing explanations, and more practical demonstrations of data entry using statistical software such as SPSS and Excel and should include practical demonstration of applying tests of significance. Participants were also recommended to provide books/e-books on statistics and research methodology.

A study done by Ponniaha et al. [12] also recommended that more explanation be provided on some topics in the BCBR course. They are training in biostatistics, particularly with regard to sampling techniques, calculating sample sizes, using data management software, and computing measures of association.

Over half of the study participants indicated that they would have understood the material better if the interactive sessions had been held throughout the course (Table 6). Incorporating interactive elements, such as live discussions or Q&A sessions, can foster deeper understanding and address learner queries in real time.

In order to evaluate eligibility for certification, this course gave both formative and summative assessments. The participants were required to finish the online, automatically graded assignments for the formative assessment following each lesson. Ten multiple-choice questions made up each assignment. The academic staff went through several internal revision cycles before coming up with a set of questions for each lecture. Before the deadline, course participants were free to turn in as many assignments as they wanted; the assignment that was turned in last was given the highest score. To be qualified for registration for the final test, an assignment needs to be completed with at least a 50% score [12].

Among study participants, 22.45% reported course assignments as difficult and very difficult, while half of the study participants said it was neither simple nor difficult to complete the course assignments (Table 7). The course designers were successful in creating assignments that are neither easy nor difficult, and such perfectly designed assignments make the learners effectively understand the course. A study conducted by Ponniaha et al. reported that assignment submission-related issues were faced by a few users while using the Android app [12].

Nearly 67.09% of the participants in our study informed us that submitting assignments with a set due date will increase the effectiveness of the course (Table 10). In contrast, according to the updated BCBR course rules, there is no fixed time frame for finishing assignments, and there is no mandatory requirement for signing up for the exam during the same course cycle.

Nearly 88.78% of study participants agreed that they did not face any technical glitches during exams (Table 9). The remaining 11.22% reported that they encountered technical difficulties such as computer glitches, internet connectivity issues, or software malfunctions during online exams. Having dedicated technical support staff available to troubleshoot and resolve these issues promptly minimizes disruptions and ensures that the candidates can focus on the exam without unnecessary stress.

Satisfaction with the assistance provided at the exam center was found to be 66.58% (Table 8). The remaining 27.55% faced issues due to a lack of proper assistance and guidance. Availability of assistance and guidance is essential for maintaining fairness, reducing candidate anxiety, safeguarding exam integrity, and continuously improving the examination process. Prioritizing and improving both technical support and assistance offered at exam centers can ensure a positive experience for all candidates.

In our study, the difficulty of the exam was found to be neither easy nor difficult by 48.21%, indicating that questions were relatable to the course material (Table 12). Conversely, 24.23% of participants found the exam challenging, suggesting areas where additional support or clarification may be needed in the curriculum.

After completion of the course, 89.03% of the study participants informed that their ability to conduct research studies was enhanced (Table 14). This finding ultimately reflects the success of the course.

Limitation of study

A possible limitation of the study's generalizability to other educational levels or professional groupings is its preponderance of postgraduate students. The study's reliance on self-reported data for perceived difficulty, satisfaction with assistance during exams, and effectiveness of the course may introduce response bias, where participants might overstate or understate their experiences based on social desirability or personal biases.

## Conclusions

The BCBR course equips postgraduate students and teaching faculty with a broad range of skills necessary to do outstanding research, advance their careers, and make a major contribution to their fields.

This course facilitated its participants to improve their ability to conduct research. By conducting this study, we found that refinement of the course material should be done to improve the effectiveness of the course. Adding the following materials such as more practical demonstrations of data entry using statistical software such as SPSS, Excel, and others including a practical demonstration of applying tests of significance and providing books/e-books on statistics and research methodology might help participants improve their knowledge and upskill their ability to conduct research.
